# Methionine-Capped Gold Nanoclusters as a Fluorescence-Enhanced Probe for Cadmium(II) Sensing

**DOI:** 10.3390/s18020658

**Published:** 2018-02-23

**Authors:** Yan Peng, Maomao Wang, Xiaoxia Wu, Fu Wang, Lang Liu

**Affiliations:** 1 Laboratory of Environmental Sciences and Technology, Xinjiang Technical Institute of Physics & Chemistry, Chinese Academy of Sciences, Urumqi 830011, Xinjiang, China; yanpeng9236@sina.com (Y.P.); wma8899@sina.com (M.W.); wuxx@ms.xjb.ac.cn (X.W.); 2 College of Chemistry and Chemical Engineering, Xinjiang University, Urumqi 830046, Xinjiang, China

**Keywords:** methionine, gold nanoclusters, fluorescence, enhancing, cadmium ion, water, milk

## Abstract

Gold nanoclusters (Au NCs) have been considered as novel heavy metal ions sensors due to their ultrafine size, photo-stability and excellent fluorescent properties. In this study, a green and facile method was developed for the preparation of fluorescent water-soluble gold nanoclusters with methionine as a stabilizer. The nanoclusters emit orange fluorescence with excitation/emission peaks at 420/565 nm and a quantum yield of about 1.46%. The fluorescence of the Au NCs is selectively and sensitively enhanced by addition of Cd(II) ions attributed to the Cd(II) ion-induced aggregation of nanoclusters. This finding was further used to design a fluorometric method for the determination of Cd(II) ions, which had a linear response in the concentration range from 50 nM to 35 μM and a detection limit of 12.25 nM. The practicality of the nanoprobe was validated in various environmental water samples and milk powder samples, with a fairly satisfactory recovery percent.

## 1. Introduction

Heavy metal ions are prevalent in agriculture, industry and drinking water, causing serious environmental problems. Specifically, cadmium ions (Cd^2+^), which exist widely in air, soil, and water [1], are extremely toxic, not only causing serious environmental and health problems, but also being listed by the U.S. Environmental Protection Agency (EPA) as one of 126 priority pollutants [2]. Several methods have therefore been established to detect Cd^2+^ ion, such as atomic absorption spectrometry (AAS) [3], atomic fluorescence spectrometry (AFS) [4] and inductively coupled plasma–atomic emission spectroscopy (ICP–AES) [5]. Though they have acceptable sensitivity and selectivity, the operating conditions are normally cumbersome and costly, which inherently limits their wider application. Therefore, it is critical to explore a low-cost and simple method for the determination of Cd^2+^ in real samples.

In recent years, fluorescent sensors have attracted much attention in the detection of Cd^2+^ due to their excellent properties of easy operation, high sensitivity, selectivity, and real-time monitoring [6,7,8,9]. In the past few decades, a great variety of fluorescence probes have been reported for the determination of Cd^2+^, including organic dyes [10,11,12,13] and quantum dots (QDs) [14,15,16,17]. However, those QDs are limited by the potential leakage of heavy metal elements, where the organic dyes suffer from small Stokes shifts and poor photo-stability [18,19]. Consequently, developing alternative and environmentally friendly materials is of great significance.

Noble metal nanoclusters (NCs), as a star in the family of metal nanomaterials, are gradually coming into view [20]. Owing to their ultrafine size (usually less than 2 nm) [21], which is equivalent to the electronic Fermi wavelength, NCs possess the nature of molecules, including discrete energy level, strong light luminescence, good light stability, biocompatibility and other unique physical and chemical properties, thus exhibiting great potential in the field of sensing and imaging [22,23,24]. The use of NCs to detect Cd^2+^ has been studied by several groups. In 2016, Niu et al., developed a dumbbell-shaped CQDs/Au NCs nanohybrid as a ratiometric fluorescent sensor for Cd^2+^ through a “turn-off” method [25]. In 2017, Naaz and Chowdhury synthesized a photoluminescent Ag NCs through fine tuning of sunlight and ultrasound to detect thiophilic metal ions (including Cd^2+^ ion), which is also based on fluorescence quenching [26]. However, such “turn-off” modes inevitably produce false results, which is not preferable in practice because other quenchers or environmental stimulus may also cause fluorescence quenching, and thus affect the sensitivity and authenticity of the test [27]. Although considerable progress has been made, finding new rapid and efficient nanoprobes to selectively recognize Cd^2+^ is still of great importance.

**Figure 1:**
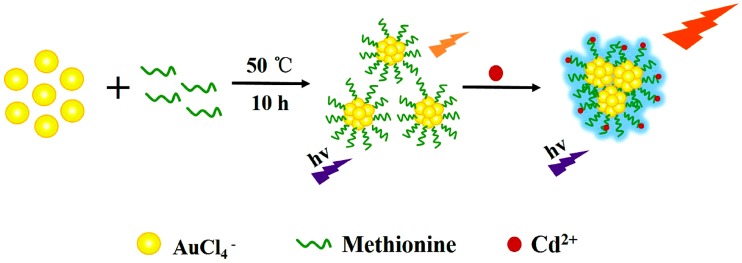
Schematic illustration of the Gold nanoclusters’ (Au NCs) formation and the Cd^2+^ induced fluorescence enhancing of Au NCs.

Herein, we demonstrated a green and facile strategy to prepare water-soluble, stable orange-emitting gold nanoclusters by using methionine as a stabilizer. The presence of Cd^2+^ ions leads to the aggregation of nanoclusters with enhancement of fluorescence intensity (Figure 1). Moreover, the nanoprobe was successfully applied to detect Cd^2+^ in various real samples with impressive efficiency and satisfactory recovery, showing great potential in practical application.

## 2. Materials and Methods

### 2.1. Chemicals and Reagents

Chloroauric Acid (HAuCl_4_·3H_2_O, 99.9%), DL-methionine (C_5_H_11_NO_2_S, 99.9%) were obtained from Adamas-beta and Ascorbic Acid (C_6_H_8_O_6_, 99.9%) was obtained from Sigma-Aldrich (Shanghai, China). All competitive metal ions and anions used in selectivity testing were acquired from Sinopharm Chemical Reagent Co. Ltd. (Shanghai, China). All the reagents were used as received without further purification. Ultrapure water (resistivity: 18.2 MΩ·cm) was obtained from a Millipore purification system (Shanghai, China).

### 2.2. Instruments

UV-vis absorption spectra were recorded on a Shimadzu UV-1800 spectrophotometer (Kyoto, Japan). Fluorescence spectra were performed on a Hitachi F-7000 fluorescence spectrometer (Tokyo, Japan). X-ray photoelectron spectroscopy (XPS) measurements were carried out using an ESCALAB 250Xi spectrometer (Thermo Fisher Scientific, Waltham, MA, USA). High-resolution transmission electron microscopy (HR-TEM) and energy-dispersive X-ray spectroscopy (EDX) data were obtained on a FEI Tecnai G2 F20 S-TWIN transmission electron microscopy instrument operating at 200 kV (Hillsboro, OR, USA). Electrospray ionization mass spectrometry (ESI-MS) measurements were conducted on a QSTAR elite liquid chromatography-mass spectrometry (LCMS, SCIEX, Toronto, ON, Canada), equipped with a common ESI source. Time-resolved luminescence intensity decay was recorded on a Horiba JY Fluorolog-3 molecule fluorometer (Paris, France), and samples were excited by a 375 nm laser light source. Inductively coupled plasma-optical emission spectrometry (ICP-OES) data were obtained on VISTA-PRO CCD Simultaneous ICP-OES (Varian, Palo Alto, CA, USA). Dynamic light scattering (DLS) was determined using a Nano-ZS90 (Malvern Instruments, Malvern, UK).

### 2.3. Synthesis of Au NCs

All glassware used in the following procedures was thoroughly cleaned with freshly prepared aqua regia (3:1 conc. HCl/HNO_3_ *v*/*v*) and rinsed with ultrapure water prior to use. In a typical procedure, 3.88 mL of HAuCl_4_ aqueous solution (2.5 mM) was mixed with 8 mL of methionine solution (80 mM) and 1.2 mL of NaOH (0.5 M) for 30 min, and then 3 mL of L-ascorbic acid (20 mM) was added into the solution at 50 °C within 9.5 h, obtaining yellow solutions. Afterwards, the reaction solution was centrifuged at 8000 rpm for 10 min to discard large particles and dialyzed with water via a dialysis membrane (1000 Da) for 48 h to remove the free ions and ligand. The resulting solution was stored in the dark at 4 °C for use. At the same time, the solid powder can be obtained by freeze-drying.

### 2.4. Fluorescent Detection of Cd^2+^

CdCl_2_·5/2H_2_O was used for the study of Cd^2+^ detection. A 10 mM stock solution of CdCl_2_·5/2H_2_O was prepared, from which various Cd^2+^ concentration were prepared by serial dilution. The fluorescence spectra were recorded to observe the fluorescence intensity change in the presence of Cd^2+^ at different concentrations. To evaluate the sensitivity toward Cd^2+^, 100 µL of Cd^2+^ solution of various concentrations was added into 1.9 mL of the prepared Au NCs solutions, and the mixtures were incubated at room temperature for the specified time before spectral measurements. The fluorescence spectra were recorded at room temperature with the maximum excitation wavelength at 420 nm; both the excitation and emission slit widths were 5 nm.

### 2.5. Selectivity Measurement

To check the selectivity of Au NCs, a series of competitive metal ions and anions with the identical concentration of 1 mM (100 µL), including Na^+^, K^+^, Mg^2+^, Ba^2+^, Pb^2+^, Ca^2+^, Al^3+^, Cr^3+^, Fe^3+^, Sn^2+^, Cl^−^, H_2_PO_4_^−^, Br^−^, SCN^−^, NO_3_^−^, CO_3_^−^, IO_3_^−^, SO_4_^2−^, NO_2_^−^, I^−^ and ClO_4_^−^, were respectively introduced to a group of Au NCs solutions (1.9 mL) to measure the change of fluorescence intensity. The resulting solutions were studied by fluorescence spectra at room temperature with excitation wavelength at 420 nm, both the excitation and emission slit widths were 5 nm. For enhancing efficiency measurement, the change of fluorescence (F/F_0_) was determined by comparing the intensity of the fluorescence emission of different solutions. F_0_ represents the fluorescence intensity of the Au NCs in the absence of metal ions, and F is the fluorescence intensity of the Au NCs in the presence of different concentrations of Cd^2+^ or different interfering ions.

### 2.6. Analysis of Real Samples

To evaluate the applicability of the Au NCs to real samples, tap water, lake water, milk powders and camel milk powders were spiked with Cd^2+^ and tested in the assay. Tap water was collected from our laboratory (Urumqi, China) and river water from Hong Lake (Urumqi, China). All water samples were freshly collected and filtered with 0.45 μm micropore membrane prior to analysis. Milk powders and camel milk powders were purchased from different food manufacturers and pretreated according to the previous Huang’s report [28]. The obtained sample filtrates were adjusted the pH to 8, then analyzed with the proposed strategy and ICP-OES method. A recovery test was performed by spiking the pre-treated samples with the standard solutions of Cd^2+^ ions (3, 5 and 10 µM) and subsequently analyzing them by the aforesaid procedure.

## 3. Results and Discussion

### 3.1. Characterization of Au NCs

**Figure 2:**
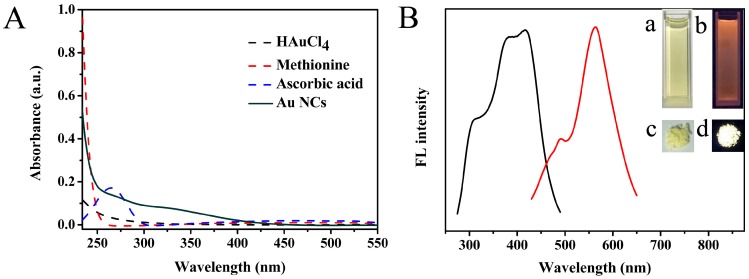
UV–vis absorption spectra (**A**) and fluorescence excitation (black) and emission (red) spectra (**B**) of the as-synthesized Au NCs. The insets of B show photographs of the Au NC aqueous solution in room light (a) and UV light (b), and powder in room light (c) and UV light (d).

In the present work, we chose methionine as a stabilizer and chloroauric acid (HAuCl_4_·3H_2_O) as metal precursor to prepare Au NCs. The synthetic conditions were systematically optimized, as depicted in Figure S1; the fluorescence intensity of the Au NCs reached its maximum when the concentration of methionine was 80 mM, NaOH was 0.5 M, and ascorbic acid was 20 mM, reaction at 50 °C within 10 h. The spectral characteristics of Au NCs were studied by UV–Vis and luminescence spectrometer. As shown in Figure 2A, the spectrum of as-prepared Au NCs displays insignificant absorption peaks, and does not have the characteristic surface plasma resonance (SPR) peak of larger Au NPs (normally around 520 nm). As previously reported, the ultra-small sizes of methionine-stabilized Au NCs demonstrated molecular-like properties, which were different from relatively large-sized metal nanoparticles [29]. From Figure 2B, it can clearly be observed that the as-prepared Au NCs exhibit strong fluorescence emission at 565 nm with maximum excitation at 420 nm. The Stokes shift of the Au NCs was calculated to be 145 nm. A Stokes shift of less than 150 nm can be attributed to interband transitions of electrons in the Au NCs [30]. The fluorescence emission spectra of Au NCs upon excitation in the range of 360–460 nm showed constant peaks (Figure S2), indicating that the optical signal was actual luminescence from the Au NCs, rather than light scattering [31]. The as-prepared methionine-stabilized Au NCs were highly dispersed in aqueous solution, and exhibited obvious yellow suspension in the room light (inset a of Figure 2B) and emitted intense orange fluorescence (inset b of Figure 2B) under UV light irradiation with the excitation at 365 nm. In addition, a solid powder could be obtained by freeze-drying, which also appeared bright orange in room light (inset c of Figure 2B) and showed an intense yellow fluorescence (inset d of Figure 2B) under UV light. Furthermore, the quantum yield of as-prepared Au NCs was calculated to be about 1.46% (rhodamine B in ethanol was used as reference), which is comparable to other NCs prepared using amino acid as stabilizer [32,33]. Moreover, there was no obvious change in the position of the emission peak of Au NCs after storing in the dark at 4 °C for three months and quenching of the fluorescence intensity (Figure S3), indicating that the Au NCs are suitable for long-term use.

**Figure 3:**
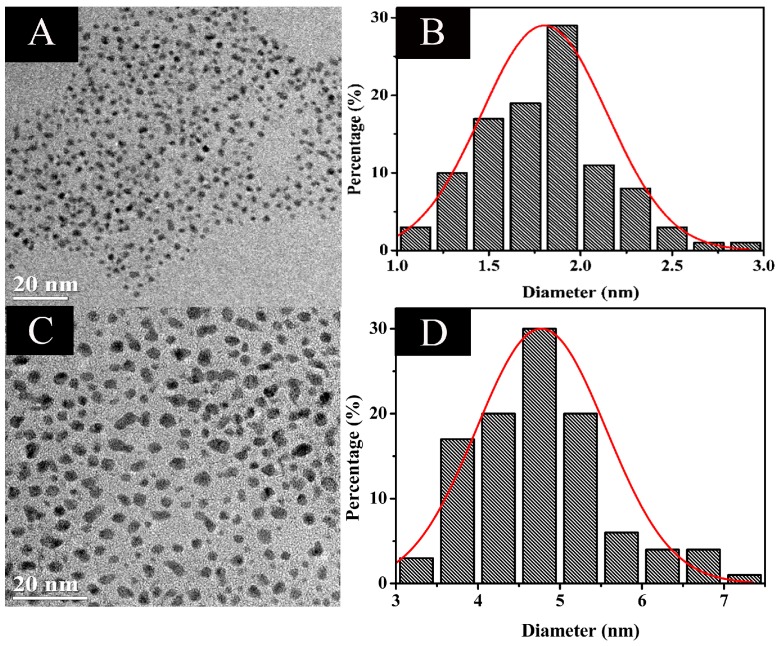
Representative high resolution transmission electron microscopy (HRTEM) images of the as-synthesized luminescent Au NCs (**A**); the particle-size distribution histogram of Au NCs (**B**); Au NCs in the presence of 50 µM Cd^2+^ (**C**); and particle-size distribution histogram (**D**).

The morphology and size distribution of the Au NCs were observed by HR-TEM. Figure 3A,B shows the typical TEM images of the Au NCs and their size ranges, it can be seen that the size of samples is less than 2 nm, and no aggregation appears. The hydrodynamic diameter of Au NCs measured using DLS was approximately 4.24 nm (Figure S7), which further verified the successful synthesis of ultrasmall Au NCs. In addition, the elemental composition of the Au NCs can be confirmed by EDX. From Figure S4 and Table S1, the results indicate that the atomic content of Au in the sample is of 47.10%.

**Figure 4:**
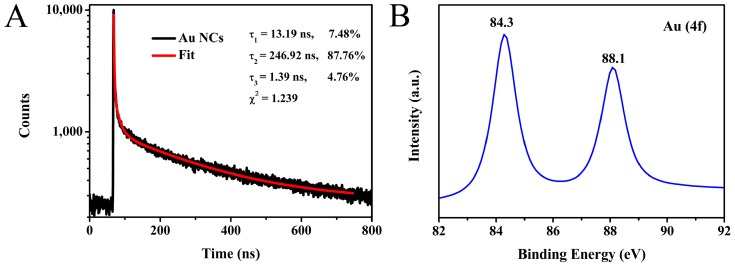
Fluorescence lifetimes of Au NCs in aqueous solution (**A**); data were collected at 565 nm with excitation at 375 nm. X-ray photoelectron spectroscopy (XPS) spectra of Au 4f of Au NCs (**B**).

Furthermore, the fluorescence decay response of the prepared Au NCs showed three components at 246.92 ns (87.76%), 13.19 ns (7.48%) and 1.39 ns (4.76%); the average fluorescence lifetime of Au NCs was calculated to be 217.74 ns (Figure 4A). As reported in previous research, the long fluorescence lifetime might result from the Au(I)-S complex structure [34], which might have the possibility for fluorescence lifetime imaging in future [35]. Moreover, XPS measurement was performed to verify the valence state of the metal elements in the prepared Au NCs. As exhibited in Figure 4B, it can be seen that the binding energy of Au 4f appeared at 84.3 and 88.1 eV, respectively, which indicated that Au(0) and Au(I) coexist in Au NCs. The Au(I) on the surface of nanoclusters plays a vital role in stabilizing the nanoclusters [36]. In order to further determine the composition of the metal core from as-synthesized Au NCs, ESI-MS was applied. The mass spectrum of Au NCs (negative mode) is displayed in Figure S5, the main charges appearing at 1003.4, 1019.4 Da are assigned to [Au_6_L_6_-2Cl]^2−^ and [Au_6_L_6_-Cl-H]^2−^ (“L” refers to “methionine”), suggesting the Au NCs are mainly composed of Au_6_ clusters.

### 3.2. Optimization of Sensing Conditions

**Figure 5:**
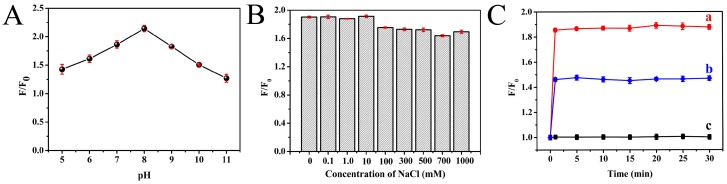
The effect of pH (**A**), concentration of NaCl (**B**) and incubation time (**C**) on fluorescence intensity of Au NCs upon addition of Cd^2+^ ions at different concentrations (35 μM (a); 10 μM (b); and 0 μM (c)), respectively.

It is interesting that the fluorescence intensity of the as-prepared Au NCs was significantly enhanced in the presence of Cd^2+^ ions. In order to obtain a highly sensitive response for the detection of Cd^2+^, the optimization of pH values, ionic strength and incubation time was carried out systematically. The pH value of the reaction solution could greatly influence the interaction between Au NCs and Cd^2+^ ions. The fluctuating pH values in the range of 4.0–11.0 were investigated. As displayed in Figure 5A, the fluorescence intensity increased significantly with the increasing solution pH, and reached its maximum when pH reached 8.0. A possible explanation for this is that the pH influences the Cd^2+^ ion speciation in solution [37]. Therefore, pH 8.0 was selected as the optimum pH value for Cd^2+^ ion detection. Meanwhile, to explore the potential application of the sensing system under high-ionic-strength environments, the stability of the Au NCs was investigated in the presence of various concentrations of NaCl (0.01 mM to 1.0 M). As shown in Figure 5B, the fluorescence intensity of Au NCs in the different salt-containing solutions remained almost unchanged compared with that in the absence of NaCl, indicating the high stability of Au NCs. In addition, the effect of reaction time on the fluorescence intensity was also studied. It can be seen from Figure 5C that the fluorescence intensity increased rapidly, and reached its maximum at around 1 min, after which it remained relatively stable. Therefore, a reaction time of 1 min was chosen in this experiment.

### 3.3. Au NCs Fluorescent Sensing of Cd^2+^

**Figure 6:**
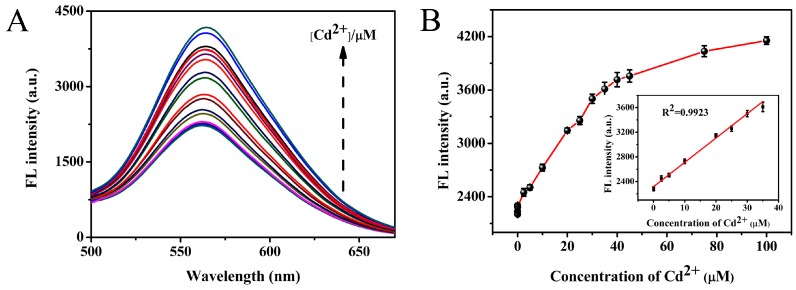
(**A**) The fluorescence response of the Au NCs in the presence of various concentrations of Cd^2+^ (0.0025, 0.005, 0.025, 0.05, 2.5, 5, 10, 15, 20, 25, 30, 35, 40, 45, 50, 75, 100 µM); (**B**) Relationship between fluorescence intensity and Cd^2+^ concentration. The inset picture shows the linear detection range for 0.05–35 µM of Cd^2+^.

Under the optimized reaction conditions, the sensing properties were examined in the presence of Cd^2+^. As shown in Figure 6A, the fluorescence intensity of Au NCs gradually increased with increasing concentration of Cd^2+^, accompanied with the color changes of the solution from faint orange to bright orange under UV irradiation (Figure S6). Moreover, Figure 6B shows the relationship between fluorescence intensity and Cd^2+^ concentration, and the inset picture represents the linear response over the Cd^2+^ concentration range from 50.0 nM to 35.0 µM; the correlation coefficient R^2^ was calculated as 0.9923, and the limit of detection (LOD) was determined to be 12.25 nM, which was lower than the maximum safety level of Cd^2+^ (ca. 44 nM) in drinking water permitted by the U.S. Environmental Protection Agency (EPA) [38]. In comparison with other previously reported methods, the proposed nanoprobe exhibits comparable or more efficient properties for Cd^2+^ detecting (Table S2).

### 3.4. Selectivity of the Detection System

**Figure 7:**
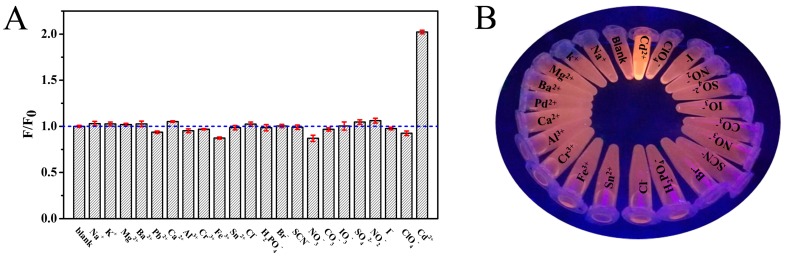
(**A**) Selective experiments of Au NCs for other competitive metal ions and anions; (**B**) Photographs of Au NCs under UV light after being incubated with various ions.

In order to investigate whether the synthesized probe is specific for Cd^2+^, the fluorescence response of this sensing system was also tested by other competitive metal cations and anions (Na^+^, K^+^, Mg^2+^, Ba^2+^, Pb^2+^, Ca^2+^, Al^3+^, Cr^3+^, Fe^3+^, Sn^2+^, Cl^−^, H_2_PO_4_^−^, Br^−^, SCN^−^, NO_3_^−^, CO_3_^−^, IO_3_^−^, SO_4_^2−^, NO_2_^−^, I^−^ and ClO_4_^−^) under the same conditions. As shown in Figure 7A, no significant increase was observed by adding other ions into the Au NCs solution, only Cd^2+^ showed a fluorescence enhancement phenomenon—about a twofold photoluminescence (PL) increment. It can be clearly seen that only the Cd^2+^-containing Au NCs solution exhibited greatly enhanced luminescence under UV light irradiation with excitation at 365 nm (Figure 7B). All these results confirm that the present fluorescent probe exhibits the high selectivity required for Cd^2+^ ion assays in real samples.

### 3.5. Mechanism of the Sensing System

The corresponding mechanism of the as-prepared Au NCs for fluorescence-enhanced Cd^2+^ sensing was also explored. In general, both the metal core and the ligand shell of the NCs can be used as recognition components, as they are able to specifically interact with analytes [22]. High resolution transmission electron microscopy (HRTEM) was used to evaluate the differences of as-prepared Au NCs in the absence and presence of Cd^2+^ ions. As displayed in Figure 3A, the Au NCs are well-dispersed irregular spherical shapes with an average size of 1.80 ± 0.34 nm (Figure 3B). After incubation with 50 μM Cd^2+^, the average size of Au NCs showed an obvious enlargement (Figure 3C) and was about 4.76 ± 0.28 nm (Figure 3D). In addition, as indicated in Figure S7, the hydrodynamic diameter of the Au NC solution obtained from DLS was 4.24 nm, and then increased to 27.63 nm after addition of 50 µM Cd^2+^ into the solution. Therefore, we speculate that the Cd^2+^ ions might link Au NCs via chelating bonds with a carboxyl group or an amino group in methionine adsorbed on the Au nanoclusters, as the ligand shell contributes many surface-related properties to the NCs [22]. However, the detailed mechanisms require further study.

### 3.6. Real Sample Analysis

To evaluate whether the fluorescent Au NCs probe is applicable to natural systems; tap water, lake water, milk power and camel milk powders were investigated. Analytical results showed that the fluorescence tests were affected in the real samples due to the complex matrix. However, the fluorescence Au NC probe exhibited excellent performance. The average recoveries of four spiked samples ranged from 95.33% to 106.21%, with RSDs of 0.51–3.56% (*n* = 3) at three spiked levels (Table 1), and these results were in good agreement with those obtained by ICP-OES, which indicated the practicality and reliability of the Au NC probe for the detection of Cd^2+^ in various samples.

## 4. Conclusions

In summary, we successfully prepared water-soluble orange-emitting Au NCs using methionine as stabilizer. The synthesis process is simple, green and environmentally friendly, without using any toxic organic reagents. The resulting Au NCs exhibited impressive properties, such as ultrafine size, long fluorescence lifetime and excellent stability. Au NCs were further used as fluorescence nanoprobe to selectively, sensitively and efficiently recognize Cd^2+^, with response times of as low as 1 min. Moreover, the sensing system is verified by detecting Cd^2+^ ions in water and milk samples, the average recoveries are in the range of 95.33% to 106.21%, suggesting this strategy may be extended to the efficient detection of Cd^2+^ in various conditions.

## Figures and Tables

**Table 1:** Analytical results for Cd^2+^ sensing in real samples.

Sample	Detected (µM)	Spiked (µM)	Found (µM)	Recovery (%)	RSD (%) (*n* = 3)	ICP-OES (µM)
Tap water	ND ^1^	3.00	2.89	96.33	2.56	2.97
		5.00	5.10	101.95	3.56	5.07
		10.00	10.62	106.21	1.45	10.08
Lake water	ND ^1^	3.00	2.86	95.33	2.37	3.02
		5.00	4.84	96.74	2.15	5.06
		10.00	10.41	104.14	0.51	10.08
Milk powders	ND ^1^	3.00	2.96	98.66	2.56	2.92
		5.00	5.06	101.29	1.68	5.03
		10.00	10.43	104.32	1.56	10.05
Camel milk powders	ND ^1^	3.00	2.87	95.67	2.79	2.95
		5.00	4.96	99.23	2.31	5.04
		10.00	10.26	102.62	1.87	10.09

^1^ Not detectable.

## Supplementary Materials

The following are available online at http://www.mdpi.com/1424-8220/18/2/658/s1, Figure S1: Optimization of synthesized conditions of Au NCs, Figure S2: Emission spectra of fluorescent Au NCs prepared in a typical synthesis with different excitation wavelengths, Figure S3: The fluorescence spectra of Met-Au NCs storing in dark at 4 °C before and after three months, Figure S4: EDX spectrum for as-prepared Au NCs, Figure S5: ESI mass spectra (negative mode) of as-synthesized Au NCs, Figure S6: The color change of Au NCs under UV irradiation when exposed to different concentration of Cd^2+^ ions, Figure S7: Hydrodynamic diameter measured using DLS of Au NCs (**A**) and Au NCs in the presence of 50 µM Cd^2+^ (**B**) at neutral pH, Table S1: Quantification Results of EDX from Au NCs, Table S2: Comparison of this method with other reported approaches for the detection of Cd^2+^ using different optical systems.
