# Supplementary material for: Methionine-Capped Gold Nanoclusters as a Fluorescence-Enhanced Probe for Cadmium(II) Sensing
Source: Sensors (Basel). 2018 Feb 23;18(2):658. doi: 10.3390/s18020658 (PMC5855495; doi:10.3390/s18020658)
Supplement: Supplementary file 1 [file sensors-18-00658-s001.pdf]

Supplementary Material

Methionine capped gold nanoclusters as a fluorescence-enhanced probe for cadmium(Ⅱ) sensing

Yan Peng [1,2], Maomao Wang [1], Xiaoxia Wu [1], Fu Wang [1*] and Lang Liu [2*]

[1] Laboratory of Environmental Sciences and Technology, Xinjiang Technical Institute of Physics & Chemistry, Chinese Academy of Sciences, Urumqi 830011, Xinjiang, China.

[2] College of Chemistry and Chemical Engineering, Xinjiang University, Urumqi 830046, Xinjiang, China.

*Corresponding Authors:

E-mail address: wangfu@ms.xjb.ac.cn; Tel.: +86-991-3835879 (F.W.)

E-mail address: liulang@xju.edu.cn; Tel.: +86-991-8588883 (L.L.)

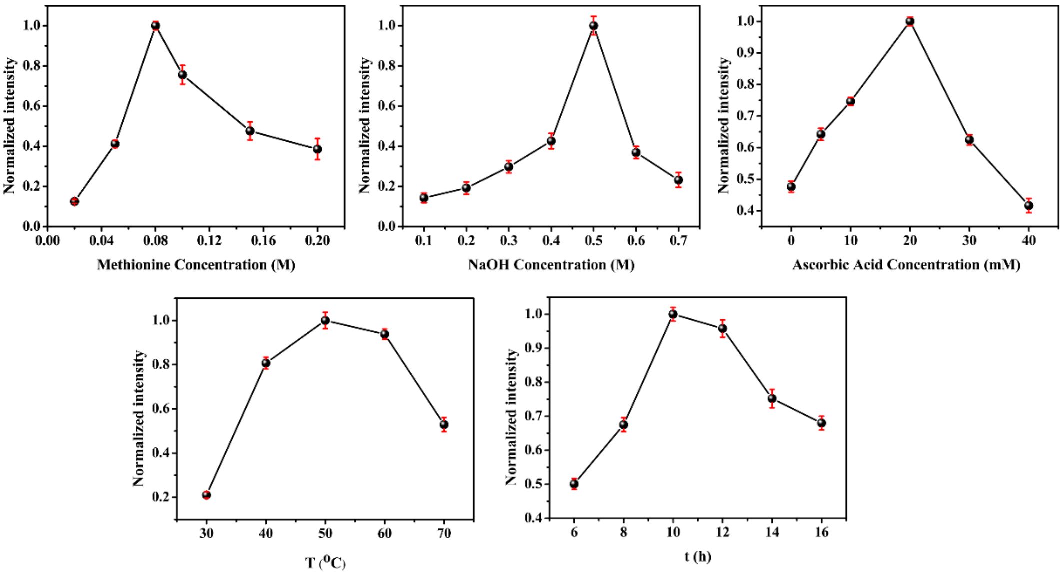

Figure S1. Optimization of synthesized conditions of Au NCs

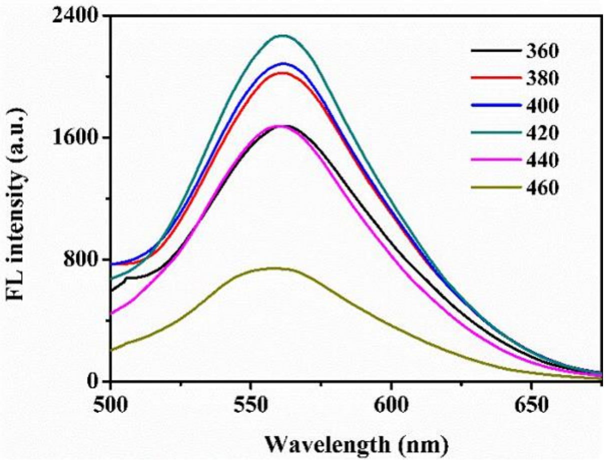

Figure S2. Emission spectra of fluorescent Au NCs prepared in a typical synthesis with different excitation wavelengths.

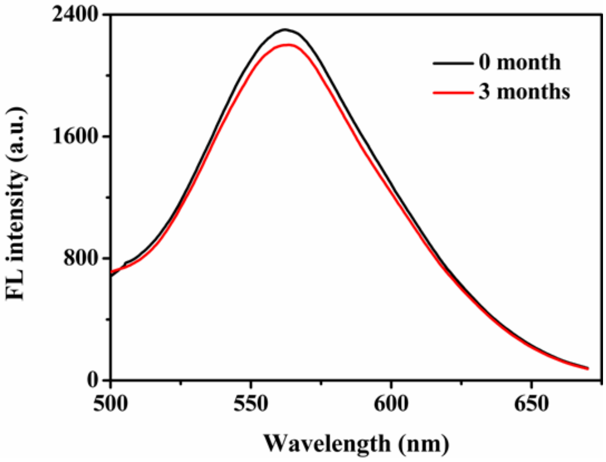

Figure S3. The fluorescence spectra of Met-Au NCs storing in dark at 4℃ before and after three months.

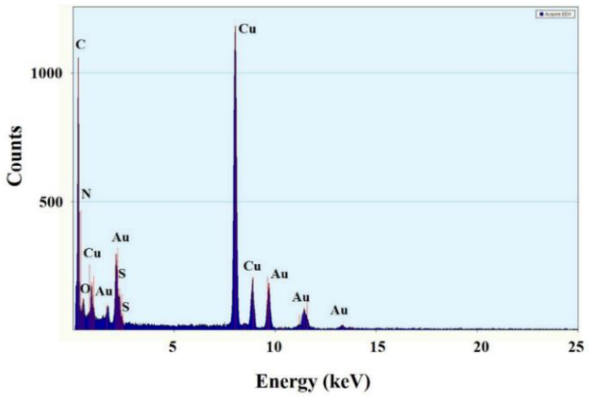

Figure S4. EDX spectrum for as-prepared Au NCs.

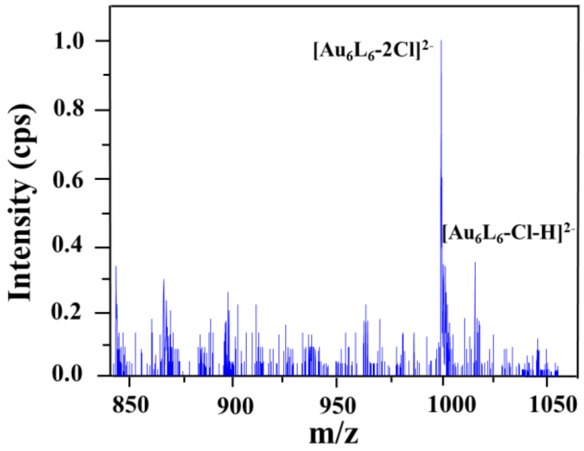

Figure S5. ESI mass spectra (negative mode) of as-synthesized Au NCs

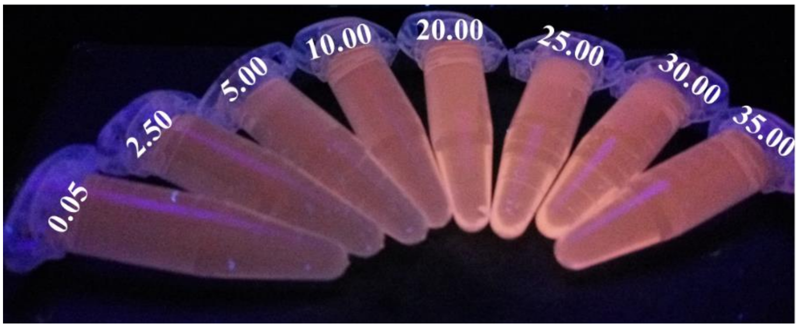

Figure S6. The color change of Au NCs under UV irradiation when exposed to different concentration of Cd^{2+} ions.

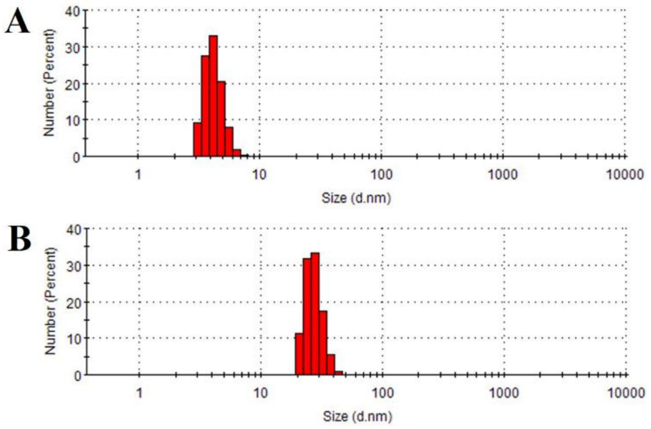

Figure S7. Hydrodynamic diameter measured using DLS of Au NCs (A) and Au NCs in the presence of 50 μM Cd^{2+} (B) at neutral pH.

Table S1. Quantification Results of EDX from Au NCs.

	Element	Weight%	Atomic%	Uncert%	Detector Correction	K-Factor
Au NCs	N(K)	1.08	7.22	0.24	0.28	3.466
	O(K)	6.08	35.33	0.32	0.51	1.889
	S(K)	5.64	16.34	0.25	0.93	1.021
	Au(k)	87.17	41.10	1.33	0.99	4.256

Table S2. Comparison of this method with other reported approaches for the detection of Cd^{2+} using different optical systems.

Probe	LOD (M)	Linear range (M)	Time	Reals samples	Reference
Colorimetric (Ag NPs)	1.30×10^{-7}	2.27×10^{-7}-3.18×10^{-6}	10 min	Water	[1]
Aptamer probe (Cd-2-2)	4.0×10^{-8}	0-1.0×10^{-6}	30 min	-	[2]
Organic dyes (NHQ)	2.165×10^{-7}	0-6×10^{-6}	-	-	[3]
Organic dyes (PD)	3.20×10^{-8}	0-2.5×10^{-6}	10 min	Cells	[4]
QDs (SiO_2-S-Zn-CdS)	1.0×10^{-7}	3.0×10^{-7}-2.6×10^{-5}	7 min	solanum nigrum L	[5]
QDs (Ag_2S)	5.46×10^{-7}	1.0×10^{-6}-4.0×10^{-5}	2 min	Water	[6]
NCs (BSA-Au/Ni))	1.75×10^{-9}	5.0×10^{-9}-1.0×10^{-6}	10 min	serum	[7]
NCs (GSH-Au)	2.40×10^{-8}	0-1.4×10^{-6}	-	Water	[8]
NCs (Met-Au)	1.23×10^{-8}	5×10^{-8}-3.5×10^{-5}	1 min	Water, Milk	This work

References

1. Dong, Y.; Ding , L.; Jin, X.; Zhu, N. Silver nanoparticles capped with chalcon carboxylic acid as a probe for colorimetric determination of cadmium(II). Microchim. Acta 2017, 184, 3357–3362.
2. Wang, H.; Cheng, H.; Wang, J.; Xu, L.; Chen, H.; Pei, R. Selection and characterization of DNA aptamers for the development of light-up biosensor to detect Cd(II). Talanta 2016, 154, 498–503.
3. Xu, Z.; Li, G.; Ren, Y.Y.; Huang, H.; Wen, X.; Xu, Q.; Fan, X.; Huang, Z.; Huang, J.; Xu, L. A selective fluorescent probe for the detection of Cd2+ in different buffer solutions and water. Dalton Trans. 2016, 45, 12087–12093.
4. Huang, W.-B.; Gu, W.; Huang, H.-X.; Wang, J.-B.; Shen, W.-X.; Lv, Y.-Y.; Shen, J. A porphyrin-based fluorescent probe for optical detection of toxic Cd2+ ion in aqueous solution and living cells. Dyes Pigment. 2017, 143, 427–435.
5. Luo, X.; Wu, W.; Deng, F.; Chen, D.; Luo, S.; Au, C. Quantum dot-based turn-on fluorescent probe for imaging intracellular zinc(II) and cadmium(II) ions. Microchim. Acta 2014, 181, 1361–1367.
6. Wu, Q.; Zhou, M.; Shi, J.; Li, Q.; Yang, M.; Zhang, Z. Synthesis of water-soluble Ag2S quantum dots with fluorescence in the second near-infrared window for turn-on detection of Zn(II) and Cd(II). Anal. Chem. 2017, 89, 6616–6623.
7. Wang, Z.-X.; Guo, Y.-X.; Ding, S.-N. Fluorometric determination of cadmium(II) and mercury(II) using nanoclusters consisting of a gold-nickel alloy. Microchim. Acta 2015, 182, 2223–2231.
8. Huang, P.; Li, S.; Gao, N.; Wu, F. Toward selective, sensitive, and discriminative detection of Hg2+ and Cd2+ via pH-modulated surface chemistry of glutathione-capped gold nanoclusters. Analyst 2015, 140, 7313–7321.
